# Design and Testing of an Experimental Steam-Induced Burn Model in Rats

**DOI:** 10.1155/2017/9878109

**Published:** 2017-10-12

**Authors:** Vlad Porumb, Alexandru Florentin Trandabăț, Cristina Terinte, Irina Draga Căruntu, Elena Porumb-Andrese, Mihail Gabriel Dimofte, Dragoş Pieptu

**Affiliations:** ^1^Department of Surgery, School of Medicine, Grigore T. Popa University of Medicine and Pharmacy, 16 University Street, 700115 Iaşi, Romania; ^2^Second Surgery Clinic, Regional Institute of Oncology, 2-4 General Henri Mathias Berthelot Street, 700483 Iaşi, Romania; ^3^Department of Electrical Measurements and Materials, School of Electrical Engineering, Gheorghe Asachi Technical University, 3 Dimitrie Mangeron Street, 700050 Iaşi, Romania; ^4^Department of Pathology, Regional Institute of Oncology, 2-4 General Henri Mathias Berthelot Street, 700483 Iaşi, Romania; ^5^Department of Morpho-Functional Sciences I-Histology, School of Medicine, Grigore T. Popa University of Medicine and Pharmacy, 16 University Street, 700115 Iaşi, Romania; ^6^Department of Dermatology, School of Medicine, Grigore T. Popa University of Medicine and Pharmacy, 16 University Street, 700115 Iaşi, Romania; ^7^Department of Plastic Surgery, School of Medicine, Grigore T. Popa University of Medicine and Pharmacy, 16 University Street, 700115 Iaşi, Romania

## Abstract

**Background:**

Most of the current models for experimental burns pose difficulties in ensuring consistency and standardization.

**Aim of Study:**

We aimed to develop an automated, reproducible technique for experimental burns using steam-based heat transfer.

**Methods:**

The system developed for steam exposure was based on a novel, integrated, computer-controlled design. Three groups of rats were exposed to steam for 1, 3, and 7 seconds. The lesions were evaluated after 20 minutes, 48 hours, and 72 hours after burn induction.

**Results:**

One-second steam application produced a superficial second-degree burn; three-second application induced deep second-degree burn; and seven-second application led to a third-degree burn.

**Conclusion:**

The high level of automation of our integrated, computer-controlled system makes the difference between our system and other models, by ensuring the control of the duration of exposure, temperature, and pressure and eliminating as many potential human generated errors as possible. The automated system can accurately reproduce specific types of burns, according to histological assessment. This model could generate the reproducible data needed in the study of burn pathology and in order to assess new treatments.

## 1. Introduction

The establishment of new approaches for experimental burns is an important tool in advancing the understanding of burn pathology. Adequate methods of experimental testing using different burn models were crucial to the development of novel treatments. Rat models have been used since the 1960s and have since then evolved to better represent and mimic human burn pathology [1]. Walker and Mason (1968) established the first straightforward and reproducible burn model in 1968, based on the production of burn on rats by direct contact with hot water [2]. The rat burn model can provide useful information on how human tissues react under specific conditions due to the similarity of the tissue structure and physiology between rats and humans. Data from rat models can be extrapolated to include post-burn physiopathology due to the similarity of both tissues, rat and human, in the burn and healing processes [3].

Burns are generally created using one of four different methods in animal models: hot water, hot metal tools, electricity, and heated paraffin [3–6]. The different burn models require specific parameters such as elevated temperatures, duration of exposure, and specific materials like hot water or aluminum previously heated in a hot water bath [7–10]. Other materials can be used in a rat model burn experiment, such as a hot metal plate or an electric coil; however, these materials are more likely to cause third-degree burns due to high temperatures reaching 170°C and 400°C, respectively [8, 9].

Although burn experiments using rat models yield data that may be useful in understanding burn pathology and physiology, it is difficult to consistently induce a certain type of burn because the various parameters are often difficult to reproduce consistently within and among applications [11–19]. The aim of this study is to create a model that will allow the objective collection of data in order to study burn pathology. The reproducible model will have strictly controlled variables such as exposure time to the heat, depth of the lesion, and source of the heat.

## 2. Materials and Methods

### 2.1. Experimental Design

This study was approved on January 21, 2015, by Grigore T. Popa University of Medicine and Pharmacy Research Ethics Committee under full compliance with the European Directive 206/27.04.2004. The study included 54 Wistar male rats, each having a weight of 300 ± 20 grams. Rats were housed in individually ventilated cages with 12/12-hour light/dark cycles, food and water ad libitum. The rats were divided into three equal groups, each containing 18 rats, and labeled Group 1, Group 2, and Group 3, respectively. Group 1 rats were exposed to hot steam for one minute, Group 2 rats were exposed for three seconds, and Group 3 rats were exposed for seven seconds. The rats were anaesthetized with Ketamine/Xylazine cocktail 0.1 mL/100 g of rat, intraperitoneally. Following anesthesia, the dorsa of the rats were shaved and depilated using hair removal cream (Farmec SA, Romania). Post-burn analgesia was provided by subcutaneous administration of buprenorphine 0.05 mg/kg three times daily (Bupredine Multidose 0.3 mg/ml Produlab Pharma B. V. Holland) for 7 days or until sacrifice, if scheduled earlier than 7 days. Throughout the experiment, the rat's general state was observed and we could see normal behaviors including normal exploratory behavior, walking, standing on their hind legs, stretching upright, burrowing, and nesting with no apparent indication of pain or suffering, like puffed out hair, pinched in sides, or weight loss. After the burn induction, skin changes based on the following aspects: swelling, redness, blistering, crust, secretion, granulation tissue, bleeding, scar tissue, and local complications or infections were documented by standardized digital photography imaging. Each group was further divided into three subgroups comprised of three rats each.

### 2.2. The Integrated Steam Application System

**Figure 1:**
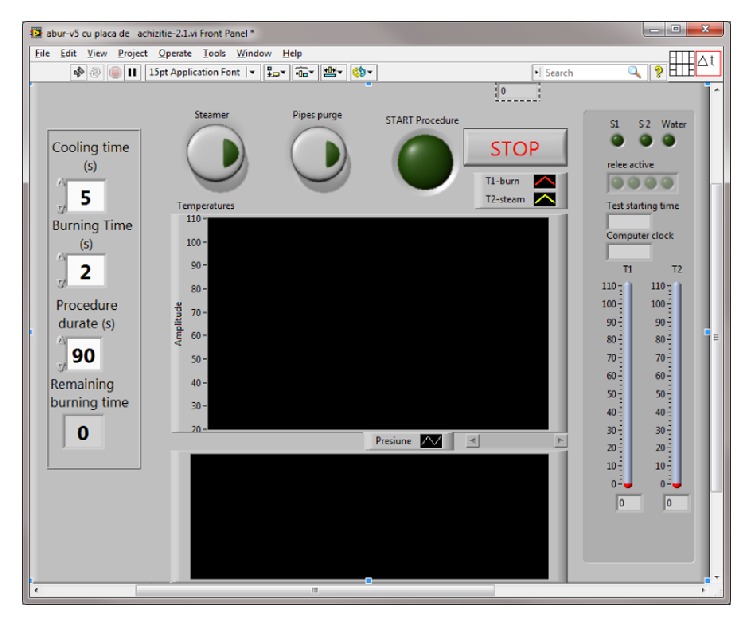
The main panel of the program's interface.

The integrated steam application system is comprised of the following:

- A computer regulates parameter control through a user interface (Figure 1) utilizing Lab VIEW 2010 software to enable the display of collected data, input, the status of the steam generator, valve activation and electrical relay, time exposure, temperatures along the steam circuit, the distance between steam nozzle and dermis, and the depth to which the steam pervaded the skin.
- The data board, which can carry out programmed computer functions, was connected to temperature (LM35) and pressure sensors, electrical relays and valves (220 Volts), a water pump, a steam generator, an electrical 12 Volt power supply, steam pipes, and silicon steam nozzle (located between the steam jet and tegument).

**Figure 2:**
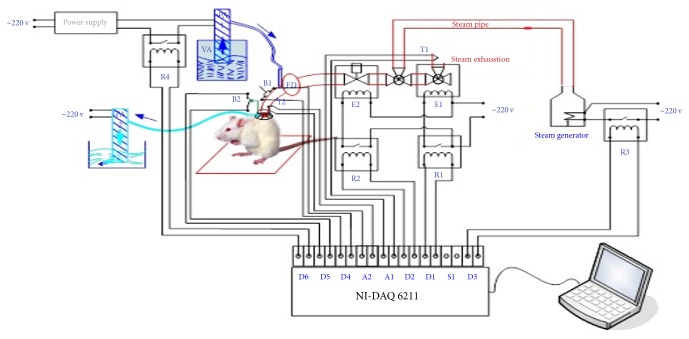
Schematic diagram of the standardized experimental model used to induce cutaneous burns in rats (R1, R2, R3, R4 - 5 Volt relays; E1, E2 - 220 V solenoid valves; T1, T2 - LM35 temperature sensors; PA1, PA2 - water pump; FD - filter sludge trap; B1 - cocking button; B2 – trigger button; NI-DAQ 6211 acquisition card with the following ports: A1 - analog input of temperature sensor T1, A2 - analog input of temperature sensor T2, D1 - digital output controlling relay R1 solenoid valve command E1, D2 - digital output controlling relay R2 solenoid valve command E2, D3 - digital output controlling steam generator, D4- digital input arming button B1, D5 - digital input trigger button B2, D6 - digital output controlling water pump command PA1; steam generator; laptop; 12 Volt power supply; steam pipe; silicone head).

The data board controls the relays and electric valves, which turn water into steam using the steam generator. The temperature of the steam is regulated by the data board and is monitored by temperature sensors. The schematic diagram of the integrated system for producing standardized burns is represented in Figure 2.

Firstly, the digital port D3 activated the steam generator producing hot steam through relay R3. Once the circuit is initiated, the acquisition card which controls the D1 port sends a signal to relay R1, activating the E1 solenoid valve, through which the steam from the steam generator is propelled. To ensure a uniform and standardized temperature of the causal burn agent, the temperature of the steam is monitored using the T1 temperature sensor.

The temperature of the steam was required to be greater than 90°C. If, however, the system was initiated accidentally, the human operator was alerted both visually and acoustically, not allowing initiation of the circuit. When the automated system is cocked, the first solenoid E1 valve is open, while the second solenoid E2 valve is closed. This prevents accidents and possibly lower temperatures that could occur if the steam would circulate within an appurtenant heat circuit.

The steam is oriented toward the silicon head by programming the T1 temperature sensor to detect temperatures greater than 90°C which allows the first solenoid E1 valve to close and the second solenoid E2 valve to open when button B1 is pressed.

Human error was avoided by creating a controlled time loop. Thus, skin steam exposure could only take place for a certain period of time. Time of exposure was calculated from the moment the silicon head touched the rat's skin via a mechanical sensor attached to a chronometer. Once the set time of steam exposure was reached, an automated mechanism closed the second solenoid E2 valve while opening the first solenoid E1 valve, blocking the nozzle through which the steam was expelled. The water pump is then activated and directed cold water for a predefined amount of time towards the same point where heat stimulus occurred.

The duration of steam exposure was controlled due to a chronometer programmed at intervals of one second, three and seven seconds, after which the automated electric valves were deactivated, ending the heat exposure period.

The temperature of the steam was recorded and monitored by a temperature sensor on the rat's skin.

The silicon head was equipped with a special device that collects hot water droplets in order not to burn the surrounding skin via direct hot water droplets.

The time of exposure and the temperature of the steam were regulated by sensors within the circuit. The steam's temperature on the skin was 94°C ± 2°C. The information collected by the sensors was automatically stored in a  .cvs file. The pressure exerted on the skin, monitored by a pressure sensor, remained constant throughout every trial. Using the Meeh-DuBois formula for surface area prediction, the burned surface represented 1% of the rat's skin, with the burn diameter being 20 mm and thus constant (represented by the number 10) *∗* weight^2/3^ = burned surface area, *K* [20].

The heat transfer version of Newton's law (*Q*_thermal energy_ = *h*_heat transfer coefficient_*A*_heat transfer surface area_(*T*_steam temp_ − *T*_skin temp_)) requires a constant heat transfer coefficient that in thermodynamics is the proportionality constant between the heat flux and the thermodynamic driving force for the flow of heat. Knowing that thermal energy is the power propagated with time application, this means that the value of our heat transfer coefficient was 5,3 W/cm2K.

### 2.3. Evaluation of Morphological Changes following Steam Application

#### 2.3.1. Macroscopic Examination

The evaluation of macroscopic transformations following steam exposure was conducted at different time intervals: 20 minutes, 48 hours, 72 hours, 7 days, 14 days, and 21 days, respectively.

The morphologic elements analyzed were color, consistency, and border (contour aspect). Following each evaluation, photos of the lesions were taken for further use in comparative evaluation.

#### 2.3.2. Microscopic Examination

Rats were sacrificed at 20 minutes, 48 hours, and 72 hours post-exposure in order to study the elements of microscopic burn morphology. The rats were sacrificed according to the ethical principles of research, internationally regulated by European Directive number 63/2010 and nationally by law number 206 from May 24, 2004. Rats were euthanized by the administration of the lethal dose of 4 times the anesthetic dose of the combination anesthetic Ketamine/Xylazine in conformity with AVMA Guidelines for the Euthanasia of Animals: 2013 Edition.

After representative samples of both burned and nonburned skin were harvested from the rat, the collected tissue samples were fixed in 3.5% formaldehyde prepared in PBS (0.01 M, pH 7.2). Sections of 0.4 micrometers were cut and then stained with standard hematoxylin-eosin (HE). All fragments were processed according to the classical protocol for the histopathologic examination of paraffin-embedded samples [4]. The histological examination was performed by an experienced pathologist. The parameters assessed included cell/tissue necrosis, acute and chronic inflammatory response, vascular lesions, granular tissue (represented by fibroblasts, myofibroblasts, neovascularization and new collagen), connective tissue repair/new connective tissue (healing), and reepithelialization.

## 3. Results

### 3.1. Overall Assessment of Steam-Induced Lesions

**Figure 3:**
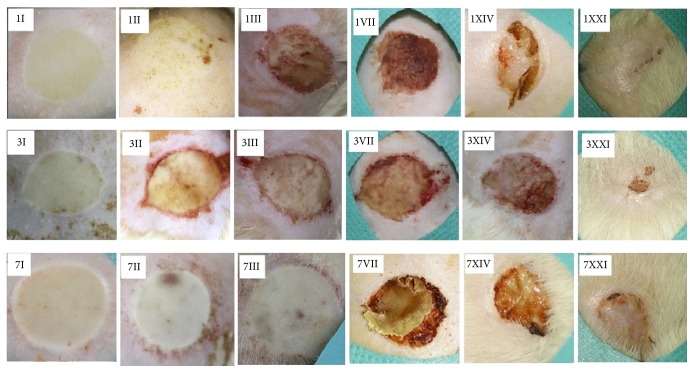
Lesions at different time intervals after steam-inflicted burns. The Arabic numerals represent the seconds of steam exposure (one second, three and seven seconds). The roman numerals represent the time intervals at which the lesions were examined once the heat was removed from the rat's skin (I = 20 minutes, II = 48 hours, III = 72 hours, VII = 7 days, XIV = 14 days, XXI = 21 days).

In each study group assessed, the burn lesions were macroscopically described as mainly uniform and homogenous with a round or slightly oval shape and a visible border (contour) separating the burned from nonburned skin (Figure 3). Moreover, the lesions healed uniformly with minimal differences noted in scab formation and detachment.

In each of the subgroups, it was observed that the histopathological changes between the three rats were similar, with minor differences concerning the degree of inflammatory infiltrate.  Table 1 summarized the key histological features at several endpoints.

### 3.2. Morphological Changes after the One-Second Steam Exposure

#### 3.2.1. Evaluation after 20 Minutes

**Figure 4:**
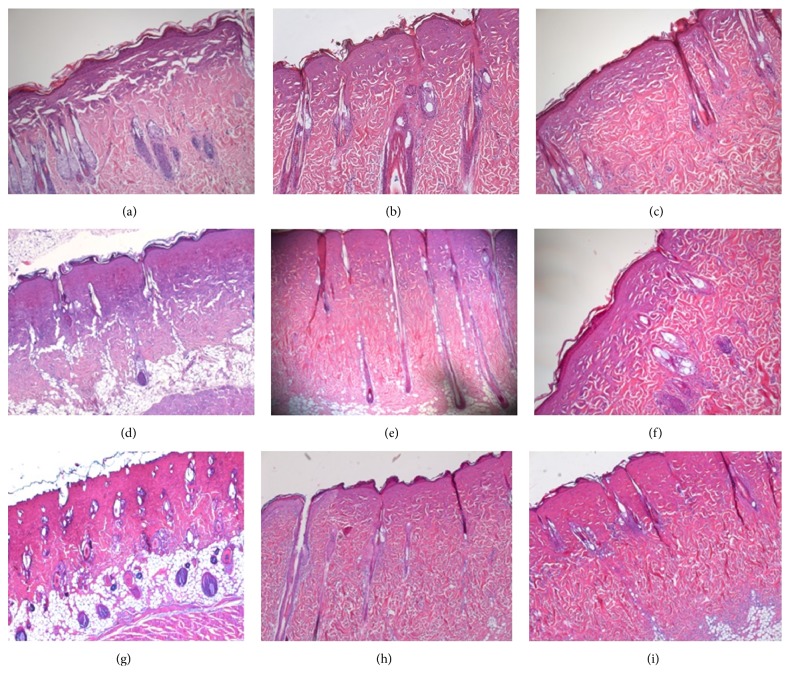
Dermal and epidermal morphological changes in a burn inflicted by (a) one-second steam exposure – assessment at 20 minutes post-burn, (b) one-second steam exposure – assessment at 48 hours post-burn, (c) one-second steam exposure – assessment at 72 hours post-burn, (d) three-second steam exposure – assessment at 20 minutes post-burn, (e) three-second steam exposure – assessment at 48 hours post-burn, (f) three-second steam exposure – assessment at 72 hours post-burn, (g) seven-second steam exposure – assessment at 20 minutes post-burn, (h) seven-second steam exposure – assessment at 48 hours post-burn, (i) seven-second steam exposure – assessment at 72 hours post-burn.

Upon evaluating the epidermis, the stratum spinosum and the stratum granulosum were homogeneous and could not be precisely identified due to the abolishment of cellular outlines and cellular shadowing. The stratum corneum was intact. The collagen in the superficial papillary dermis was homogenous, which corresponds to the presence of coagulation necrosis (Figure 4(a)).

#### 3.2.2. Evaluation after 48 Hours

Similar morphological aspects were identified within the epidermis and superficial papillary dermis in congruency with those found in the group of rats exposed to a one-second burn and the latter evaluation of the lesion 20 minutes after (Figure 4(b)).

#### 3.2.3. Evaluation after 72 Hours

In comparison to the other rat groups in which the lesions were studied at an earlier time interval, the morphological changes encountered were dominated by an abundance of inflammatory infiltrate comprised of both acute and chronic phase cells in the superficial papillary dermis (Figure 4(c)).

### 3.3. Morphological Changes after the Three-Second Steam Exposure

#### 3.3.1. Evaluation after 20 Minutes

In the epidermis, significant cell homogenization occurred between the stratum basale, stratum spinosum, and stratum granulosum due to the deletion of cellular limits, disappearance of the intercellular space, corresponding to desmosomes, and the persistence of the stratum lucidum and corneum. The coagulation necrosis present in the superficial papillary dermis continued into the upper part of deep reticular dermis. The lower area of the deep reticular dermis and the hypodermis maintained structural integrity (Figure 4(d)).

#### 3.3.2. Evaluation after 48 Hours

The homogenous epidermis presented erased boundaries between stratum basale, stratum spinosum, and stratum granulosum. On the superficial papillary dermis, a scattered expansion of the coagulation necrosis toward the upper area of the deep reticular dermis could be seen (Figure 4(e)).

#### 3.3.3. Evaluation after 72 Hours

The epidermal and dermal morphological changes were similar to the changes observed at the 48-hour evaluation mark; however, within the dermis an inflammatory infiltrate with acute phase cells was observed. A conglomeration of PMN's surrounded the hair follicles observed at the point where the deep reticular dermis met the area of coagulation necrosis in the superficial papillary dermis (Figure 4(f)).

### 3.4. Morphological Changes after the Seven-Second Steam Exposure

#### 3.4.1. Evaluation after 20 Minutes

Coagulation necrosis was identified in the epidermis, the superficial papillary dermis, and the whole deep reticular dermis. The lower area of the deep reticular dermis is characterized by the thickening and homogenization of collagen fibers; these fibers were horizontally orientated and ran parallel to the coagulation necrosis area. No clear border between these two areas could be identified. However, the adipose tissue, which corresponds to the hypodermis, did not suffer microscopic morphological changes (Figure 4(g)).

#### 3.4.2. Evaluation after 48 Hours

The specific aspect of coagulation necrosis was found in the epidermis, the superficial papillary dermis, and the whole deep reticular dermis (Figure 4(h)).

#### 3.4.3. Evaluation after 72 Hours

The presence of coagulation necrosis in the epidermis, superficial papillary dermis, and the deep reticular dermis was associated with the presence of an inflammatory infiltrate with both acute and chronic phase cells. These microabscesses were also identified around the external epithelial sheath of the sebaceous follicles from the boundary between the remaining deep reticular dermis and the preserved coagulation necrosis area (Figure 4(i)).

## 4. Discussion

### 4.1. The Significance of Steam-Induced Burns according to the Established Clinical Burn Classification

According to the burn classification, the expansion in depth of the burn lesion within the exposed tissue can be of multiple types, ranging from first-degree, second-degree – type A, second-degree – type B, and third-degree burns [21–24].

The first-degree burn is superficial and the lesion is located at the surface of the dermis. A second-degree – type A burn, also known as a partial superficial burn or a superficial dermis burn, is when the lesion is located on the surface of the epidermis and the superficial papillary dermis. Type F, also known as a partial deep burn or a deep dermis burn, is when the lesion affects the superficial papillary dermis and the upper area of the deep reticular dermis. In this type of burn, the pilosebaceous complexes situated in the lower area of the deep reticular dermis remain intact. A deep burn or a third-degree burn affects the entire thickness comprising the epidermis, dermis, hypodermis and all cutaneous annexes, and, in some cases, even the adipose tissue.

Ideally, an experimental burn model should allow the operator to evaluate morphological and histopathological lesions and tissue modifications and to be able to assess different burn types according to burn classification. Currently, there is no reliable, reproducible burn model as the literature is limited. Initially, experimental burn systems would produce deep burns. In the last 10 to 15 years, experimental burn systems have aimed to produce partially deep burns. Over 25% of modern studies do not mention burn classification. Moreover, partial superficial burns studied are rarely classified into type A or type D, which may lead to incongruent data [4, 5, 25–27]. The burn classification is based on different degrees of lesion severity represented by the histopathological modifications observed.

As shown above, applying hot steam to the skin for one second produces a partial superficial burn, a second-degree – type A burn, characterized by the histopathological modifications described such as the presence of coagulation necrosis in the epidermis and the superficial papillary dermis. Applying hot steam to the skin for three seconds produces deep partial burns consistent with second-degree – type B burns, which affects the entire epidermis, the superficial papillary dermis, and the upper area of the deep reticular dermis and the pilosebaceous complexes as noted by the presence of coagulation necrosis.

Third-degree burns are produced when coagulation necrosis reaches the hypodermis, affecting the epidermis, superficial papillary dermis, and the deep reticular dermis. As shown, applying hot steam for seven seconds to the skin can produce these lesions.

Evaluating reproducibility in an experimental burn model can be difficult and it is important to assess two elements: (i) the depth to which the burn affected the tissue and (ii) the scab formation and healing process important in regaining integrity of the affected tissue by evaluating epithelial, conjunctive and dermal cellular/structural components. Furthermore, maintaining a constant burn target area on the rat's dorsum allows for a correct classification of burn intensity affecting only the tissue layers present in the designated area and permits the comparison of burn types after different durations of steam exposure. The reproducibility of this setup favors the observation of burn intensity over a period of time.

The healing process for second-degree – type A and second-degree – type B burns produced by applying hot steam for one second or three seconds, respectively, starts around seven days post-exposure. In the deepest area of the burn lesion, young granulation tissue appears and evolves into mature granulation tissue. The formation of new conjunctive tissue structure takes place within the dermis.

### 4.2. Advantages of the Proposed Experimental Burn Model

The experimental steam burn model allows for a comparative analysis of pathological lesions produced by different durations of heat exposure and the healing progression at several time points post-exposure. This model permits the extrapolation of data based on comparing the similarities and differences at each time interval. Histopathological changes of the tissue in the burn lesions were observed along with specific mechanisms in the healing process, which were unique at the each interval. No other described experimental burn model compares time exposure to heat source, lesion depth and periodic evaluation post-burn, and healing from a macroscopic and microscopic viewpoint.

In experimental models, the animals frequently used are rats, mice, and pigs. None of the three species can be considered superior to the others, with the obtained information through their study being complementary [28].

One of the first burn models that was described in the literature was produced by using hot water on the rats' skin [2]. The use of hot water as a vector is reported in under 10% of the experimental models, while approximately 65% of the studies use metal as a means of induction having been previously heated with hot water [2–18]. This vector presents many disadvantages such as application difficulty and work safety.

However, the use of hot water could regulate the optimum contact time and temperature as well as the quantity of heat emanated. Exposure time is harder to measure because the fluid state increases the difficulty to standardize the process of elimination. Cuttle et al. developed a bottle with a bottom constructed of an elastic membrane that contains hot water [11]. When this hot water is applied on the skin it leads to a better adaptation to the skin's uneven surface, avoiding the overburn of the skin [11]. This membrane, as well as the heated metallic blocks, does not ensure the intimate adherence to the smallest uneven topographic parts of the surface of the skin. A limit of the experimental models on hot water or hot metal heated in hot water is that these vectors produce only one type of burn at a certain temperature, making the change of temperature to obtain another degree of burn necessary [8, 9, 19]. We also need to emphasize the fact that burn models based on heat transfer at the skin level, by applying a piece of hot metal or a different energy transfer, involve a different energy transfer, according to the materials used [18, 23, 24, 29, 30]. Moreover, different skin characteristics such as water content or amount of adipose tissue influence the process of heat transfer and modify the standard of the burn.

Another model described in the literature involves the hot plate and electrical coil. They are vectors that are described as having an even surface that have the disadvantage of application of extremely high temperatures (170°C and 400°C) and consecutively producing only third-degree burns [8, 9]. These vectors are easy to apply if their mass is big enough and if the thermal energy applied is sufficient and is constantly maintained on the skin. The contact with the skin is difficult to be standardized because of anatomical differences and physiological variables. Vectors exert uneven pressure on the exposed area, with greater pressure on the skin's prominent areas and less pressure at the interface between the skin and the vector.

The motivation to further study the steam burn experimental model was that steam has an advantage over other methods of heat transfer; steam does not need a transporter and can be better quantified in order to classify burn intensity. Another advantage of using steam is that once the cold water is introduced into the pipe system, the silicon head cools down rapidly, making time exposure more precise and allowing for possible standardization. However, because this study relies on an experimental burn model, lesion expansion due to prolonged heat exposure is a limitation [29].

This system functions due to a software system and data board with programmed automated functions and safeguard loop holes. The one variable controlled by the software was duration of heat exposure. The steam's temperature was difficult to control due to the interference of ambient temperature variations. The automated software allowed temperature data to be recorded in real time and permitted the system to turn off if one of the conditions specified in the program was not met. This allowed for less incongruence in the procedure.

The integrated system used in the experimental burn model is a customized design, all elements of which are in unison with the standard criteria for burn models. The system monitors the constant pressure of the heat application, time elapsed from the beginning of the experiment until the set time is reached, and variables verified by pressure and temperature sensors and applied chronometers. This design is congruent with other models [3, 31] which make note of different temperature parameters, producing varying burn intensities within the same experimental model. In the model developed by Campelo et al. electrically heated copper was used as a vector to produce a burn using a metal object [9]. An electric sensor connected to the plate at a two-millimeter distance ensured temperatures of 100°C, 150°C, and 200°C. In this model, time was kept by a chronometer and could be used at 5 minutes after being connected to electricity [9]. The hot metal was applied to the skin at constant nine seconds. However, only microscopic lesions were observed and no data as to burn intensity or depth could be provided. Similar models, based on the use of a metal vectors heated with the help of hot water, have been developed by research groups coordinated by Pereira, Crouzet, Nasiri and Selcuk [25, 30, 32–34]. One limitation of using metal to produce burns on the skin is that there is no instrument that has been used to record the exact temperature of the metal once it touches the skin. Also, there is no data as to the temperature maintained by the vector during exposure. A solution to correct this limitation is proposed by Venter et al. and Cai et al. by applying a temperature sensor at the tip of the metal vector that produces the burn which displays the temperature on a digital screen [4, 35]. Therefore our results obtained based on the tests done are a consequence of superior methods of temperature monitoring, automated on-off safeguard loop hole if experimental variables are not met and careful study of time interval marks.

Otherwise, because a human operator measures time by starting and stopping a chronometer once the silicon heat touches the rat's skin until the time interval is reached and is then removed [25, 28, 32, 36, 37], human error may intervene due to a delayed reaction. Considering that the time intervals are measured in seconds, it is harder to detect certain errors that may occur. It is important to note that, in burn experiments where a metal object is heated by hot water and then transferred and applied to the rat's skin, a three-second (±1 second) delay may occur in order to properly apply the heated vector on the rat's skin [35]. Moreover, after the application of heat vectors, the burn may evolve because of the skin cool down [29]. The major advantage of the device used in this study consists of the automatic turn off of the vector, doubled by an immediate cooling down process of the skin with the help of cool water sent through the pipe system. Additionally, the steam's temperature is directly measured as it is expelled from the silicon head and not from the heating tub as in other experimental models [28, 32, 36, 38]. Another major, beneficial advantage, in comparison to other devices [18, 20], is that because the heating element itself is not a hard surface that comes in contact with the rat's skin it ensures lesion uniformity via heat radiation from the steam.

The design and the concept behind the integrated system used simple and efficient methods to create burns that are easy to reproduce. This system allowed the classification of burn intensity by histopathological analysis of the burn lesions produced by uniform steam burns.

Due to low material investment in the burn apparatus this model is cost efficient and can further the understanding of burn types, lesions produced, and possible topical treatments for wounds produced via heat vectors.

The contribution made to furthering scientific knowledge of burn histopathology and physiopathology lies in the ingenuity of the integrated burn model system represented by the automated on/off circuit, reproducibility, and methodology upheld by established knowledge on experimental burn model criteria.

### 4.3. Pain Management

The use of analgesia in post-burning care against pain and suffering is a fundamental requirement for many laws of ethics that guide animal experimentation [39]. However in many burn studies, the use of analgesics in postoperative care is not mentioned [18]. Some authors justify the lack of use of post-burn analgesics in full thickness burn models by the fact that nerve endings in the skin are destroyed [40]. Nevertheless, some studies document the presence of hyperalgesia and allodynia even when full thickness thermal injury is induced, suggesting activation of afferent pain pathways in the regions surrounding the initial lesion, thus commanding the use of analgesia [41]. Pain management in experimental animals aims to achieve the well-being of the animal in such a manner that they will continue the daily routine so the experiment and the parameters that are observed will not be affected. Drugs can be administered subcutaneously, intramuscularly, or orally in the drinking water supplied to animals. Among the most commonly used opioids for postoperative pain in laboratory animals is buprenorphine, mainly because of its long duration of action [42].

### 4.4. Limitations

The validation of our model is applicable for the specific species, gender, age, and burn location presented (i.e., male Wistar rats, 300 g, dorsal burn injury). While generalizability of our model to other situations with the purpose to accurately mimic burn injury in patients would be highly desirable, the focus of this study was rather to overcome the inherent limitations associated with the energy delivery approach in the available models. The highly automated and nonexpensive system to achieve consistent thermal energy delivery will allow rapid and low-cost validation of burn models in other species, different gender or age and location of burn injury. Furthermore, the specific validated model we present here may be an important tool for experimental testing of various therapeutic approaches for burn injury.

## 5. Conclusions

The integrated system described here provides a useful tool for consistently inducing standardized burns. Duration of exposure, temperature, and pressure were controlled, corrected in real time, and recorded with the help of software, eliminating as many potential errors generated by the human operator as possible. The real time measurements were the key to the development of the burn model. Variables, some of which were affected by environmental inconsistencies or human error, were observed and later controlled. The integrated system allows the production of precise burns of constant depth, with histopathological profiles closely reproducing different degrees of clinical burns. The integrated system described has potential applicability in the study of burn pathology and in experimental assessment of new treatments.

## Figures and Tables

**Table 1:** Synopsis of the key histopathological changes.

Steam exposure	Time point	Necrosis depth^*∗*^	Collagenization^*∗∗*^	Inflammatory infiltrate	Vascular lesions
One second	20 min	+	Absent	Absent	Absent
	48 hours	+	Absent	Absent	Absent
	72 hours	+	Absent	Mixt	Absent

3 seconds	20 min	++	++	Acute	Absent
	48 hours	++	++	Mixt	Present
	72 hours	++	+++	Acute	Present

7 seconds	20 min	+++	+++	Acute	Present
	48 hours	+++	+++	Mixt	Present
	72 hours	+++	+++	Acute	Present

^*∗*^Necrosis depth: + = epidermis and superficial papillary dermis; ++ = epidermis, superficial papillary dermis, and upper level of deep reticular dermis; +++ = epidermis, superficial papillary dermis, and whole deep reticular dermis; ^*∗∗*^collagenization: + = superficial papillary dermis; ++ = upper level of deep reticular dermis; +++ = whole deep reticular dermis.
